# Characterisation of an ABC transporter of a resistant *Candida glabrata* clinical isolate

**DOI:** 10.1590/0074-02760170484

**Published:** 2018-02-05

**Authors:** Debora Afonso Silva Rocha, Leandro Figueira Reis de Sa, Ana Carolina Cartagenes Pinto, Maria de Lourdes Junqueira, Emiliana Mandarano da Silva, Ronaldo Mohana Borges, Antonio Ferreira-Pereira

**Affiliations:** 1 Universidade Federal do Rio de Janeiro, Instituto de Microbiologia Paulo de Goes, Laboratório de Bioquímica Microbiana, Rio de Janeiro, RJ, Brasil; 2 Universidade Federal de Juiz de Fora, Hospital Universitário, Juiz de Fora, MG, Brasil; 3 Universidade Federal do Rio de Janeiro, Instituto de Biofísica Carlos Chagas Filho, Laboratório de Genômica Estrutural, Rio de Janeiro, RJ, Brasil

**Keywords:** Candida glabrata, ABC transporters, resistance, FK506

## Abstract

**BACKGROUND:**

*Candida glabrata* ranks second in epidemiological surveillance studies, and is considered one of the main human yeast pathogens. Treatment of *Candida* infections represents a contemporary public health problem due to the limited availability of an antifungal arsenal, toxicity effects and increasing cases of resistance. *C. glabrata* presents intrinsic fluconazole resistance and is a significant concern in clinical practice and in hospital environments.

**OBJECTIVE:**

The aim of this study was to characterise the azole resistance mechanism presented by a *C. glabrata* clinical isolate from a Brazilian university hospital.

**METHODS:**

Azole susceptibility assays, chemosensitisation, flow cytometry and mass spectrometry were performed.

**FINDINGS:**

Our study demonstrated extremely high resistance to all azoles tested: fluconazole, voriconazole, posaconazole and itraconazole. This isolate was chemosensitised by FK506, a classical inhibitor of ABC transporters related to azole resistance, and Rhodamine 6G extrusion was observed. A mass spectrometry assay confirmed the ABC protein identification suggesting the probable role of efflux pumps in this resistance phenotype.

**MAIN CONCLUSIONS:**

This study emphasizes the importance of ABC proteins and their relation to the resistance mechanism in hospital environments and they may be an important target for the development of compounds able to unsettle drug extrusion.

The *Candida* genus is present as a commensal organism in approximately 25-75% of healthy individuals. However, *Candida* species are opportunist pathogens and responsible for approximately 90% of invasive infections (Pfaller & Diekema 2007). Candidiasis is a persistent infection and commonly observed in individuals infected with human immunodeficiency virus (HIV) as well as patients undergoing transplantation procedures (Yang et al. 2012, Marukutira et al. 2014). These infections are usually attributed to *Candida albicans*. Nevertheless, *Candida glabrata* ranks second in epidemiological surveillance studies, and is considered one of the main human yeast pathogens (Marukutira et al. 2014, Muadcheingka & Tantivitayakul 2015). Treatment for candidiasis is a serious problem for contemporary medicine due to: a limited arsenal of antifungal drugs, toxicity and an increasing number of resistant cases (Pfaller & Diekema 2007, Silva et al. 2012, Catalano et al. 2013). Many *C. glabrata* clinical isolates present low susceptibility to fluconazole (FCZ), a common azole used for candidiasis treatment that targets the fungal specific ergosterol biosynthetical pathway (Ostrosky-Zeichner et al. 2003, Pfaller et al. 2004). This resistance phenotype can occur due to an alteration of the target enzyme (Erg11p) by mutation or overexpression of the ERG11 gene leading to decreased drug efficacy (Moye-Rowley 2015). Furthermore, azole resistance can be enhanced by efflux pumps, such as ABC protein transporters, a primary transporter that uses ATP hydrolysis as a source of energy to transport its substrates (CgCdr1p, CgCdr2p or CgPdh1p, CgSnq2p) (Sanglard et al. 1995, Vermitsky & Edlind 2004, Torelli et al. 2008). The aim of the present study is to evaluate if ABC transporters overexpression is related to the resistance phenotype of a *C. glabrata* clinical isolate previously selected from a total of 93 *Candida* spp. samples isolated from the University Hospital of the Federal University of Juiz de Fora, Minas Gerais (MG), Brazil, due to its high azole resistance.

## MATERIALS AND METHODS

*Clinical isolates and control strains* - Three resistant and one susceptible *C. glabrata* clinical isolate were selected from ninety-three *Candida* strains due to their high/low fluconazole (FCZ) resistance (Neves-Junior et al. 2015). These clinical isolates were obtained from patients attended at the University Hospital of the Federal University of Juiz de Fora, MG, Brazil, between 2012 and 2014. The *Candida* isolates were isolated from clinical materials obtained from ambulatory patients or from patients in intensive care (ICU). These clinical materials were mainly urine, feces, catheter, blood and other secretions. The ethical use of these materials was approved by Protocol CEP-UFJF: 079-420-2010-FR:368108; CAEE: 0056.420.000-10. Five mutant strains of *Saccharomyces cerevisiae*, kindly donated by Drs Richard Cannon and Brian Monk (University of Otago - New Zealand), were used: one null mutant, deleted all the genes that encoded efflux pumps; AD/CaCDR1 *S. cerevisiae* mutant strain that overexpresses *C. albicans* ABC transporter, CaCdr1p; AD/CgCDR1 *S. cerevisiae* mutant strain that overexpresses *C. glabrata* ABC transporter, CgCdr1p; AD/CgDR2 (AD/PDH1) *S. cerevisiae* mutant strain that overexpresses *C. glabrata* ABC transporter, CgCdr2p; and AD/124567 *S. cerevisiae* mutant strain that overexpresses Pdr5p of *S. cerevisiae* (Lamping et al. 2007). ATCC 2001 *C. glabrata* was also used as control for tests using *Candida* strains.

*Yeast culture conditions - Candida* strains were grown in YPD medium (2% glucose, 2% peptone, 1% yeast extract) at 37°C under agitation, and were harvested in the exponential phase of growth. *S. cerevisiae* mutant strains were grown under the same conditions at 30°C.

*Strain identification* - Identification of the *Candida* spp. clinical isolate was carried out using MALDI Microflex LT (Bruker Daltonics, Bremen, Germany) with the formic acid extraction procedure according to the manufacturer. Briefly, a single colony of each strain grown overnight in YPD agar was suspended in 300 µL of de-ionised water and 900 µL of absolute ethanol and centrifuged at 24000 *x g* for 2 min. The supernatant was discarded and the pellet was air-dried, ressuspended in 70% formic acid and 100% acetonitrile and vortexed. The samples were centrifuged at 14462 *x g* for 2 min, and 1 µL of supernatant, in duplicate, was spotted onto a steel target and air-dried at room temperature. Before identification, each spot was overlaid with 1 µL of HCCA (α-Cyano-4-hydroxycinnamic acid, Bruker) matrix solution saturated with 50% acetonitrile and 2.5% trifluoroacetic acid, and then completely air dried. The spectra were calibrated externally using a standard calibrant ATCC *Escherichia coli* 25922, before plate identification. Raw spectra were analysed using MALDI BIOTYPER Real-time Classification software version 3.1 (Bruker Daltonik MALDI Biotyper). Strains with scores ≥ 2 were considered as reliable species identification.

*Azole susceptibility tests - In vitro* susceptibility tests were performed according to the Clinical Laboratory Standards Institute (CLSI) M27-A3 protocol by the broth microdilution assay (CLSI 2008). According to the CLSI protocol, FCZ minimal inhibitory concentration (FCZ MIC_50_) end points ≤ 8 µg/mL were categorised as susceptible (S); MIC_50_ end points between 16 and 32 µg/mL were classified as susceptible dose-dependent (SDD), and resistant (R) strains obtained MIC_50_ end points ≥ 64 µg/mL. For itraconazole (ITZ), the MIC_50_ end points ≤ 0.125 µg/mL were classified as susceptible, MIC_50_ end points between 0.25 - 0.5 µg/mL were categorised as SDD and R isolates for end points ≥ 1 µg/mL. For voriconazole (VRZ) the MIC_50_ end points ≤ 1 µg/mL were classified as S, MIC_50_ end points ≥ 2 µg/mL were categorised as SDD and R isolates for end points ≥ 4 µg/mL. For posaconazole (PSZ) 0 - 8 µg/mL concentrations were tested. Clinical breakpoints for PSZ concentrations have not been proposed yet, but the values are considered to be close to the ITZ breakpoints for *C. albicans*. Cell growth analyses were performed by microplate reader at 600 nm (Fluostar Optima, BMG Labtech, Offenburg, Germany).

*Evaluation of azole resistance reversion by FK506* - FK506 is a classical ABC transporter inhibitor (Egner & Kuchler 2000) and is used as a screening for resistance mediated by these efflux proteins. The “spot test” was used as a measure of growth as previously described by (de Sá et al. 2014). Screenings of azole resistance concentrations were previously performed in order to observe the highest azole concentrations that did not affect yeast viability. We tested 5 - 200 µg/mL of FCZ and 1 - 50 µg/mL of ITZ, 0.1 - 20 µg/mL of VRZ and 0.5 - 100 µg/mL of PSZ for clinical isolates and mutant *S. cerevisiae* strains.

For *S. cerevisiae* strains, 5 μL samples of fivefold serially diluted yeast cultures (initially suspended to an OD of 0.1) were spotted on YPD agar in 6 well sterile polystyrene plates. They were incubated in the presence of different azoles concentrations. Controls were performed using YPD alone and YPD supplemented with: azole, azole + 0.5% DMSO, azole + 10 μM FK506. Plates were incubated at 30°C for 48 h.

In the case of *C. glabrata*, the same methodology was used, but with some adaptations: 5 μL of a five-fold serial dilution from a yeast suspension containing 6 x 10^5^ cells/mL was spotted on Sabouraud agar supplemented with the compounds at 100 μM alone or combined with different azoles concentrations. The incubation of the six well plates was carried at 37°C for 48 h.

*Flow cytometry assay* - The experiment was performed using seven yeast strains: four *S. cerevisiae* mutant strains (null mutant, AD/CaCDR1, AD/CgCDR1, AD/CgCDR2), one susceptible *C. glabrata* (S) clinical isolate (code 227i), one *C. glabrata* (R) FCZ resistant clinical isolate (code 109) and one ATCC *C. glabrata* (code ATCC 2001). Then 1 x 10^3^ cells/mL were incubated overnight in 20 mL of YPD medium at 37°C under agitation. After incubation, with values varying between OD 1 to 3, the cells were centrifuged at 5000 *x g* for 5 min and washed four times with deionised water. After washing, the strains were maintained on ice for 2 h, then 6 x 10^5^ cells/mL were incubated with 5 µM of rhodamine 6G (R6G), an ABC transporter fluorescent substrate (Maesaki et al. 1999), in the presence or absence of 2% glucose for 1 h, at 37°C under agitation. The cells were centrifuged at 9000 *x g* for 2 min and washed three times with phosphate buffer saline (PBS) before analysis in duplicate by BD Accuri™ C6 Flow Cytometer to measure the R6G efflux by strains. Data analysis was conducted using Accuri™ C6 Software (Accuri Cytometers - v. 1.0.264.21).

*Preparation of plasma membranes* - Yeast plasma membrane isolates from clinical and mutant yeast strains were obtained as previously described by (Rangel et al. 2010). The plasma membrane preparations were stored in liquid nitrogen and thawed just before use.

*Sodium dodecyl sulfate-polyacrylamide gel electrophoresis (SDS-PAGE)* - Protein samples were subjected to 10% SDS-PAGE (Laemmli 1970). Protein bands were visualised by staining with Coomassie brilliant blue R-250 (Sigma). The protein concentration was measured using the Bradford assay (Bradford 1976).

*In-gel digestion* - Approximately 30 µg of total proteins obtained from membrane isolation were loaded onto 10% SDS-PAGE, followed by Coomassie Brilliant blue staining. The sample-containing band, around 160 KDa, was cut and submitted to the trypsinisation process. Each band excisions, from controls and clinical isolate samples, were de-stained with 25 mM NH_4_HCO_3_ in 50% acetonitrile for 16 h. The excised bands from the nonreduced gels were reduced using 10 mM dithiothreitol (DTT) in 25 mM NH_4_HCO_3_ solution to completely cover gel pieces and incubated for 1 h at 56°C. The samples were alkylated with 55 mM iodoacetamide in 25 mM NH_4_HCO_3_ and incubated for 45 min at room temperature in the dark. After which the solution was removed and the spots were washed with 25 mM NH_4_HCO_3_ solution in 50% acetonitrile and then dehydrated with 100% acetonitrile. A solution of 25 mM NH_4_HCO_3_ containing 100 ng trypsin gold (Promega) was added to cover the dried gel pieces. The samples were digested overnight at 37°C. The tryptic peptides were extracted from SDS-PAGE gel using 50% acetonitrile in 0.1% formic acid solution.

*Ultra-high performance liquid chromatography (UHPLC)-MS/MS mass spectrometry - electrospray ionisation (ESI)* - The extracted peptides from the SDS-PAGE gel spots were desalted on-line using a Waters Opti-Pak C18 trap column. The sample injection volume was fixed at 45 µL and the UHPLC was performed using a C18 150mm x 2mm column (SHIMADZU), eluted (300 µL/min) with a linear gradient (3-45%) of acetonitrile containing 0.1% formic acid. Electrospray tandem mass spectra were recorded using an ESI - Ion Trap spectrometer (AmaZon SL). The ESI capillary voltage was set at 4,500 V and the source temperature was 300°C. The end plate offset was 500 V. Data acquisition were conducted by TrapControl software (version 7.0, Bruker); experiments were performed by scanning from a mass-to-charge ratio *(m/z)* of 300 - 1,500 using a scan speed 8,100 *m/z* per second, applied during the whole chromatographic process. The mass spectra corresponding to each signal from the total ion current (TIC) chromatogram were averaged. Data dependent MS/MS acquisitions were performed on precursors with charge states of 2, 3 or 4 over a range of 100 - 2000 *m/z* and under a 4 *m/z* window. Collision induced dissociation (CID) MS/MS spectra were obtained using helium as the collision gas. All data were processed using Compass Data Analysis (version 1.3 SR2, Bruker).

*Mass spectrometry data analysis* - The proteins were identified by analysing the precursor and fragmentation spectra using MASCOT software (Matrix Science, version 2.1). One missed cleavage per peptide was allowed and an initial mass tolerance of 0.5 Da was used in all searches. Cysteines were assumed to be carbamidomethylated and a variable modification of methionine (oxidation) was allowed.

## RESULTS

*Clinical source and species identification* - After a screening of 93 *Candida* spp. clinical isolates provided by the University Hospital of the Federal University of Juiz de Fora, MG, Brazil, between 2012 and 2014 (Neves-Junior et al. 2015), four strains were selected: three R strains (codes 107, 109 and 211i) and one S (code 227i) to antifungal FCZ. All clinical isolates were identified as *C. glabrata* specie by MALDI-TOF mass spectrometry analysis. These three strains were selected due to the low fluconazole susceptibility observed in *C. glabrata* specie.

*Azole susceptibility* - Selected *C. glabrata* strains were previously classified as S, susceptible-dependent dose (SDD) and R to FCZ according to the CLSI M27-A3 protocol (CLSI 2008, Neves-Junior et al. 2015). In the present study, other azoles such as ITZ, PSZ and VRZ, in different concentrations, were also tested. We selected three *C. glabrata* fluconazole resistant strains that had been previously chemosensitised by FK506, a classic ABC transporter inhibitor (Neves-Junior et al. 2015). As observed in Table I, only *C. glabrata* presented a high resistant phenotype to all azoles tested: MIC_50_ breakpoints were higher than the last concentration tested in this study. According to the CLSI-M27-A3 protocol, 109 strain was also considered resistant to ITZ (≥ 1 µg/mL) and VRZ (≥ 4 µg/mL). This clinical isolate presented cell growth at the maximum PSZ concentration tested (≥ 8 µg/mL). This *C. glabrata* strain (code 109) was selected for further experiments due to its high resistant profile in comparison to the other FCZ resistant clinical isolates: 107 and 211i. The FCZ susceptible clinical isolate (code 227i) and the ATCC *C. glabrata* strain (ATCC 2001 *C. glabrata)* were also considered susceptible to all azoles tested.

**TABLE I:** Minimum inhibitory concentration (MIC_50_) of resistant and susceptible to fluconazole (FCZ), itraconazole (ITZ), voriconazole (VRZ) and posaconazole (PSZ) *Candida glabrata* isolates according to the CLSI M27-A3 protocol

Species	Code	*MIC_50_ FCZ	MIC_50_ ITZ	MIC_5_VRZ	MIC_5_ PSZ
*C. glabrata* (R)	109	> 1000	> 2	> 8	> 8
*C. glabrata* (R)	107	> 1000	> 1	< 0.5	> 8
*C. glabrata* (R)	211i	> 250	> 2	< 1	< 1
*C. glabrata* (S)	227i	< 8	< 0.125	< 0.250	< 1
*C. glabrata*	ATCC 2001	< 8	< 1	< 0.5	< 0.5

* values in µg/mL; R: resistant; S: susceptible.

**Fig. 1:**
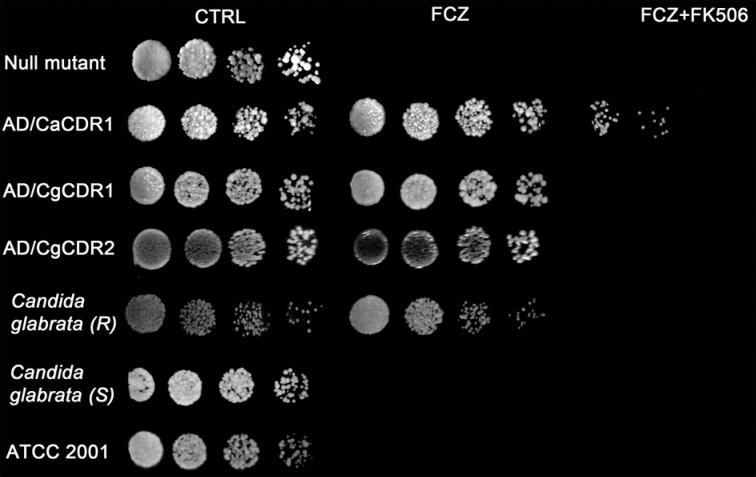
chemosensitisation assay using FK506 in the presence or absence of fluconazole (FCZ). *Saccharomyces cerevisiae* mutant cells were used as control: null mutant, AD/CaCDR1, AD/CgCDR1 and AD/CgCDR2 (CgPdh1). Different FCZ concentrations were used in each strain: null mutant, *Candida glabrata* (S), C., ATCC2001 8 µg/mL; AD/CaCDR1 and AD/CgCDR1 160 µg/mL; AD/CgCDR2 20 µg/mL; *C. glabrata* (R) 100 µg/mL. FK506 (10 µM).

*Reversion of azole resistance* - The chemosensitisation assay using FK506 and different azoles was performed in order to evaluate the possible resistance mechanism of the 109 strain. *S. cerevisiae* mutant strains were also used as controls (Fig. 1, Table II). Several concentrations of FCZ, ITZ, VRZ and PSZ were tested in solid YPD (*S. cerevisiae*) or Sabouraud (*Candida* isolates) medium and none of them affected cell growth (data not shown). Each azole was used in different concentrations in combination with FK506 according to each specie and strain. For this test, It was selected the concentration of azole that did not affect cell viability. Resistant strains presented resistance phenotype reverted after incubation with FK506 and FCZ. Null mutant, a *S. cerevisiae* mutant strain that does not present any efflux pumps proteins, was susceptible to low doses of FCZ (8 µg/mL). Similar susceptibility was observed in clinical isolate *C. glabrata* (S) and ATCC 2001 for FCZ in the same concentration.

**TABLE II:** Summary of chemosensitisation assays using FK506 in the presence or absence of different azoles: itraconazole (ITZ), voriconazole (VRZ) and posaconazole (PSZ). The positive and negative signals indicate growth or absence of cell growth, respectively. FK506 (10 µM)

	Null mutant	AD/CaCDR1	AD/CgCDR1	AD/CgCDR2	*Candida glabrata* (R)	*C. glabrata* (S)	*C. glabrata* ATCC2001
CTRL	+	+	+	+	+	+	+
ITZ	-	+	+	+	+	-	-
	(1 µg/mL)	(2 µg/mL)	(25 µg/mL)	(25 µg/mL)	(100 µg/mL)	(1 µg/mL)	(1 µg/mL)
ITZ+FK	-	-	-	-	-	-	-
CTRL	+	+	+	+	+	+	+
VRZ	-	+	+	+	+	-	-
	(0.1 µg/mL)	(2 µg/mL)	(2 µg/mL)	(0.1 µg/mL)	(10 µg/mL)	(0.1 µg/mL)	(0.1 µg/mL)
VRZ+FK	-	-	-	-	-	-	-
CTRL	+	+	+	+	+	+	+
PSZ	-	+	+	+	+	-	-
	(0.5 µg/mL)	(0.5 µg/mL)	(2 µg/mL)	(0.5 µg/mL)	(100 µg/mL)	(0.5 µg/mL)	(0.5 µg/mL)
PSZ+FK	-	-	-	-	-	-	-

Table II provides a summary of several chemosensitisation assays using different azoles (ITZ, VRZ, PSZ). Note that the null mutant strain, 227i strain and *C. glabrata* ATCC2001 were susceptible to the lowest azole concentration used, as a consequence, the azole combination with FK506 did not lead to cell growth (-). For resistant mutant strains (AD/CaCDR1, AD/CgCDR1, AD/CgCDR2) and 109 clinical isolate, the maximum concentration tested in previous screening did not affect cell growth, meanwhile, this same concentration in combination with FK506 provoked cell death (Table II).

**Fig. 2:**
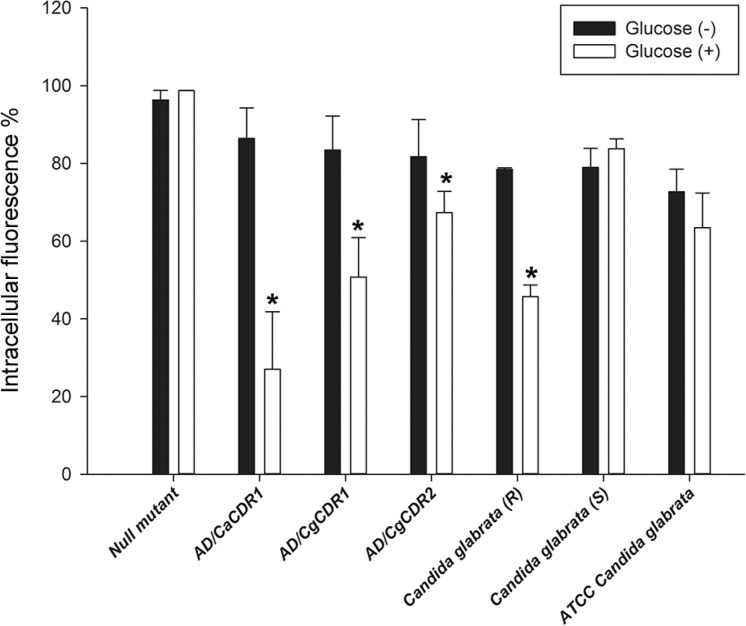
cytometry efflux assay of *Saccharomyces cerevisiae* controls (null mutant, AD/CaCDR1, AD/CgCDR1 or AD/CgCDR2), *Candida glabrata* resistant (R) and susceptible (S), ATCC 2001 *Candida glabrata* strains using R6G as fluorescent substrate in the presence and absence of glucose. The results are the average of the experiments performed in triplicate. Dark blue bar = no glucose; Light blue bar = with glucose. (*): p < 0,05.

*Flow cytometry analysis* - A flow cytometry assay was performed in order to observe the extrusion of R6G, a fluorescent substrate of ABC transporters, by resistant and susceptible strains. Four *S. cerevisiae* strains were used as controls: one susceptible (null mutant) and three FCZ resistant (AD/CaCDR1, AD/CgCDR1, AD/CgC-DR2) (Fig. 2). The *S. cerevisiae* FCZ resistant controls were able to pump out R6G in the presence of glucose while the null mutant presented approximately 96% of intracellular fluorescence in the absence and presence of glucose. The 109 clinical isolate extruded R6G significantly: in the absence of glucose (-), the intracellular accumulation of R6G was 78%, and in the presence of glucose (+) only 46% was fluorescent. A similar significant extrusion was observed in *S. cerevisiae* AD/CgCDR1 (glucose (-) 84% of fluorescence and glucose (+), 51%). It is important to note that susceptible controls 227i strain and ATCC *C. glabrata* did not extrude the fluorescent probe significantly after glucose incubation. These results confirm those obtained by the chemosensitisation assay once reverted strain pumped out R6G (Fig. 2).

**Fig. 3:**
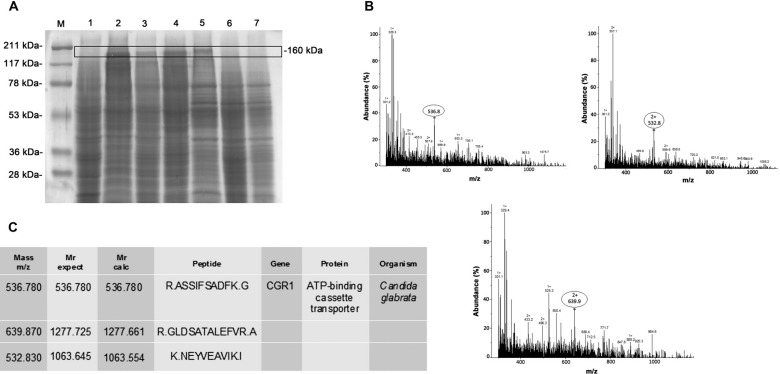
identification of the ABC transporter peptides from *Candida glabrata* (R) clinical isolate by electrospray ionisation (ESI)-MS/MS. (A) Plasma membrane preparations of control *Saccharomyces cerevisiae* mutant strains and clinical isolates separated by sodium dodecyl sulfate-polyacrylamide gel electrophoresis (SDS-PAGE) as follows: 1- null mutant; 2- AD/124567 (Pdr5p ABC transporter of *S. cerevisiae*) (+); 3- AD/CgCDR1 (CgCdr1p of *C. glabrata*); 4- AD/CgCDR2 (CgCdr2p of *C. glabrata*); 5- *C. glabrata* (R); 6- *C. glabrata* (S) and 7- *C. glabrata* ATCC2001. Excised bands correspond to molecular weight of 160kDa (ABC transporters). (B) ESI-MS/MS analysis identified peaks corresponding to three different peptides in an ABC transporter protein. (C) Mascot algorithm analysis of three peptides led to the identification of a *C. glabrata* ABC transporter gene, CgCDR1.

*UHPLC-MS/MS mass spectrometry - ESI* - MS analysis was performed in order to identify the ABC transporter protein in susceptible strains samples (null mutant, 227i strain and *C. glabrata* ATCC2001) and azole resistant *S. cerevisiae* strains (AD/CgCDR1, AD/CgCDR2 and 109 strain). Another *S. cerevisiae* mutant strain (AD/124567) overexpressing its own ABC transporter, Pdr5p, was used as control in this experiment. Plasma membrane preparation of each yeast strain was separated by SDS PAGE (Fig. 3A). As expected, the ABC transporter of null mutant (lane 1) was not visualised by SDS-PAGE neither the MS/MS analysis. ABC proteins were observed in all the resistant mutants and clinical strains (lanes 2, 3, 4 and 5, approximately 160 kDa). It is worth mentioning that the susceptible clinical strain (227i isolate, lane 6) and *C. glabrata* ATCC2001 (lane 7) did not present a detectable band. ESI-MS/MS analysis of the excised band from the 109 strain plasma membrane preparation resulted in three different *m/z* peaks of 536.8 (corresponding to a charge of +1), 532.8 (corresponding to a charge of +2) and 639.9 (corresponding to a charge of +2) (Fig. 3B). The identified peptides were R. ASSIFSADFK. G, K. NEYVEAVIK. I and R. GLDSATALEFVR. A. The Mascot analysis of these three peptides identified the ABC transporter protein CgCdr1 (Fig. 3C). The excised band from controls, AD/124567 (lane 2), AD/CgCDR1 (lane 3) and AD/CgCDR2 (lane 4), were analysed by ESI- MS/MS and corresponded to more than 10 peptides of the ABC transporter proteins (data not shown) (Fig. 3).

## DISCUSSION

*C. glabrata* is the second most common cause of mucosal and blood-stream candidiasis, it ranks in epidemiological surveillance studies after *C. albicans* and it is considered as one of the main human yeast pathogens (Pfaller & Diekema 2007, Muadcheingka & Tantivitayakul 2015). Azoles, mainly FCZ, are by far the most commonly used antifungal drug in clinical practice but, in previous decades, there have been many cases of resistance to antifungal agents used in the prophylaxis and treatment of *Candida* species infections (Almeida et al. 2013, Jiang et al. 2013).

*C. glabrata* usually presents low FCZ susceptibility, the most common azole used in candidiasis treatment (8-27% of isolates demonstrate a MIC_50_ ≥ 64 µg/mL) (Ostrosky-Zeichner et al. 2003, Pfaller et al. 2004, Paul & Moye-Rowley 2014). Studies have demonstrated that *C. glabrata* drug resistance occurs as a result of its haploid nature as well as the ability of rapid mutation after exposure to azole agents (Safdar et al. 2002). This resistance phenotype can occur due to an alteration of the target enzyme by the mutations/overexpression of its encoding gene or by overexpression of ABC transporters genes (CgCDR1, CgCDR2 or CgPDH1 and CgSNQ2). Increased gene expression leads to a higher protein level of these efflux pumps decreasing intracellular drug concentration and as a consequence leads to resistance phenotype, accompanying broad range drug tolerance (Sanglard et al. 1995, Sanguinetti et al. 2005, Torelli et al. 2008, Morschhauser 2010). In this study, we analysed whether the mechanism of resistance of the *C. glabrata* clinical strain, isolated from blood samples, is correlated to an ABC transporter overexpression. Thus *S. cerevisiae* mutant strains overexpressing CaCDR1, CgCDR1 CgCDR2 genes and a null mutant as the susceptible control were used. Moreover, one susceptible *C. glabrata* clinical isolate which was collected from tracheal secretion was also used.

For the susceptibility assays, in the present study we observed that the *C. glabrata* (R) clinical isolate was resistant to all azoles tested, including PSZ and VRZ, according to the CLSI M27-A3 protocol (Table I). Several studies have demonstrated that there has been an increase of antifungal resistance cases for treatment and prophylaxis infections related to *Candida* spp. over the last few decades. (Almeida et al. 2013, Jiang et al. 2013). *C. glabrata* presents a greater propensity to develop azole resistance probably due to molecular mechanisms as increased and/or mutation expression of genes encoding enzymes responsible for ergosterol biosynthesis; or this resistance can be enhanced by efflux pumps such as ABC proteins, a primary transporter responsible for active drug extrusion (Sanglard et al. 1995, Vermitsky & Edlind 2004, Torelli et al. 2008, Ge et al. 2010).

In order to evaluate the possible contribution of ABC transporters overexpression on resistance phenotype of this clinical strain (code 109), we performed a chemosensitisation test using FK506, a classic ABC transporter inhibitor, in combination with different azoles (Egner & Kuchler 2000). This result demonstrated that different azoles (FCZ, ITZ, VRZ and PSZ) in combination with FK506 reverted the resistance phenotype of all resistant strains tested in the present study (*S. cerevisiae* mutant strains: AD/CaCDR1, AD/CgCDR1 and AD/CgCDR2 and 109 clinical isolate) (Fig. 2, Table II). This outcome was expected for *S. cerevisiae* mutant strains since these isolates overexpress ABC transporters encoding genes. The chemosensitisation results of the 109 clinical isolate suggest that the overexpression of this efflux pumps is due to FK506 reversion.

Furthermore, a flow cytometry assay using R6G, a fluorescent substrate of ABC transporters, demonstrated that the 109 strain pumped out the fluorescent stain (46% of intracellular fluorescence, glucose (+), similarly to *S. cerevisiae* strain AD/CgCDR1 (51% of intracellular fluorescence) suggesting the overexpression of these efflux pumps.

The identification of ABC proteins was confirmed by plasma membrane isolation, followed by SDS-PAGE and finally, ESI-MS/MS analysis. In lanes 2-5 (Fig. 3) a detectable band with an approximate molecular weight of 160 KDa that referred to ABC proteins according to identification by MS/MS was observed. The 109 clinical isolate (lane 5) excised band resulted in three different *m/z* peaks: 536.8 (+1), 532.8 (+2) and 639.9 (+2) where three peptides were identified: R. ASSIFSADFK. G, K. NEYVEAVIK. I and R. GLDSATALEFVR. A and which the Mascot algorithm software analysis identified as an ABC transporter protein, gene CgCDR1 of the *C. glabrata* specie. These data confirm the presence of efflux proteins and their involvement in resistance phenotype of a *C. glabrata* clinical isolate extremely resistant to higher concentrations of all azoles tested.

Previous studies have shown increased expression levels of ABC efflux pump genes in *C. glabrata* and their involvement in azole-resistant isolates of *C. glabrata* in hospital environments (Miyazaki et al. 1998, Sanglard et al. 2001, Sanguinetti et al. 2005). Our study emphasizes the importance to study this efflux pump due to its correlation to high antifungal resistance in clinical isolates in developing countries. The high incidence of resistance phenotype in *C. glabrata* clinical isolates reveals the urgent need to develop compounds responsible to inhibit or unsettle these ABC proteins in order to prevent drug extrusion and as consequence a resistance profile.
